# High-Reliability Perovskite Quantum Dots Using Atomic Layer Deposition Passivation for Novel Photonic Applications

**DOI:** 10.3390/nano12234140

**Published:** 2022-11-23

**Authors:** Tzu-Yi Lee, Tsau-Hua Hsieh, Wen-Chien Miao, Konthoujam James Singh, Yiming Li, Chang-Ching Tu, Fang-Chung Chen, Wen-Chung Lu, Hao-Chung Kuo

**Affiliations:** 1 Department of Photonics, College of Electrical and Computer Engineering, National Yang Ming Chiao Tung University, Hsinchu 30010, Taiwan; 2 Technology Development Center, InnoLux Corporation, Hsinchu 35053, Taiwan; 3 Institute of Communications Engineering, College of Electrical and Computer Engineering, National Yang Ming Chiao Tung University, Hsinchu 30010, Taiwan; 4 Semiconductor Research Center, Hon Hai Research Institute, Taipei 11492, Taiwan; 5 Executive Office, SkyTech Institute, Hsinchu 303, Taiwan

**Keywords:** ALD passivation technology, perovskite quantum dots, reliability, micro-displays, VLC

## Abstract

In this study, we propose highly stable perovskite quantum dots (PQDs) coated with Al_2_O_3_ using atomic layer deposition (ALD) passivation technology. This passivation layer effectively protects the QDs from moisture infiltration and oxidation as well as from high temperatures and any changes in the material characteristics. They exhibit excellent wavelength stability and reliability in terms of current variation tests, long-term light aging tests, and temperature/humidity tests (60°/90%). A white-light system has been fabricated by integrating a micro-LED and red phosphor exhibiting a high data transmission rate of 1 Gbit/s. These results suggest that PeQDs treated with ALD passivation protection offer promising prospects in full-color micro-displays and high-speed visible-light communication (VLC) applications.

## 1. Introduction

Quantum dots (QDs) have received a lot of attention in recent years as one of the prospective light emitters for wide color gamut displays that can effectively meet the demands for a conveniently tunable wavelength, high efficiency, process reliability, operating stability, and color purity [1,2,3,4]. The excellent facile and economical solution processability of colloidal QDs makes it possible to integrate inorganic semiconductors into high-performance and flexible device architectures. Because of their exceptional qualities, including their narrow emission bandwidth, high fluorescent quantum yield, tunable emission color, and wide color gamut, QDs are the leading candidate for the field of next-generation solid-state lighting and displays. QDs have recently gained considerable interest as a promising contender for a wide variety of optoelectronic applications, including light-emitting diodes (LED) [1,5], solar cells [6,7], photodetectors [8], lasers [9], photocatalysis [10], biomedical imaging [11], lighting [12], and visible-light communication (VLC) [13,14]. The commercialization of QDs in the display sector is attributed, in particular, to conventional CdSe QDs from the II-VI group, which exhibit a high photoluminescence quantum yield (PLQY) in addition to other exceptional optical properties. With tremendous success, Cd QD-based LEDs have achieved significant advancements in their ability to serve as either the active layer or color conversion layer in EL devices. However, Cd-based QDs have a limited potential for use in commercial LEDs due to their toxicity and health risks, which raises some environmental concerns when it comes to mass manufacturing for commercial use. As a result, the development of environmentally friendly counterparts has long been a crucial requirement for the future generation of displays.

Perovskite quantum dots (PQDs) are a viable contender as a replacement for Cd-based QDs in next-generation display technology owing to their near-unity PLQY, narrow emission, high quantum yield, tunable emission spectra, and short radiative lifetime. Furthermore, the mid-gap trap states are not significantly impacted by the dangling bonds on the surfaces of PQDs, which is extremely advantageous for effective radiative emissions [1,2,3,4]. It has been conclusively demonstrated that it is possible to optimize a variety of useful optical properties of PQDs, including emission wavelengths, optical absorption, long carrier diffusion lengths [15,16], and high carrier mobility [17,18], using a variety of techniques, including a straightforward ligand-mediated approach. As a result, the distinct optoelectronic properties of PQDs and their simple synthetic methods enable unprecedented advancements in contemporary optoelectronic and photovoltaic systems. Recently, PQDs have gained a considerable amount of interest in VLC applications owing to their short carrier lifetime [14,19]. There have been numerous reports on PQDs realizing white-light systems by integrating blue micro-LEDs, achieving a high modulation bandwidth for VLC [13,14]. However, PQDs typically experience considerable issues with chemical, optical, or thermal instability induced by their ionic structure and large surface energy; hence, long-term stability continues to be a barrier to their usage on an industrial scale [20,21]. Additionally, when it comes to on-chip QD LEDs, the high energy radiation from blue LEDs might cause the thermal quenching and photodegradation of PQDs, which limits their practical application. Due to these challenges, on-chip PQD LEDs have substantially lower luminous efficiency than conventional phosphor-based LEDs. As a result, increasing the stability of PQDs has become a prominent topic in recent research to extend the lifetime of QDs. Over the past few years, a number of techniques have been proposed to improve the stability of PQDs, including codoping or hybridizing with other cations, surface passivation, and so on [6,7,22].

Another approach to enhancing PQD stability is encapsulation, which involves coating or isolating the PQDs with the introduction of layers of stable organic molecules or an inorganic matrix that is both airtight and mechanically robust. Effective encapsulations can increase their resistance to oxygen or humidity by isolating PQDs from one another using an additional matrix [13,23,24,25,26,27,28,29]. In particular, the encapsulating technique may effectively protect PQDs from agglomeration in the solution and prevent anion exchange when the PQDs are mixed with various halide compositions. It can also reduce degradation when subjected to polar solvents, harsh environments, high-energy radiation, and high temperatures. The coating methods and materials commonly used today include polymers, SiO_2_, Al_2_O_3_, covalent organic frameworks (COF), etc. However, polymer and SiO_2_ coatings often require the use of highly polarized solvents [30,31,32]. After the coating is completed, the reliability can be improved, but the PLQY will decrease more. Al_2_O_3_ is mostly used in CsPbBr_3_ PQD LED devices [33,34]. The ALD passivation protection technique is used to grow a thin layer of Al_2_O_3_ on the QD element, but due to the insulating nature of Al_2_O_3_, the thick intermediate layer will block the QD element. However, due to the insulating nature of Al_2_O_3_, an excessively thick intermediate layer can block electron transfer. Therefore, the thickness is limited. COF is an advanced class of highly crystalline porous polymeric materials that are often used as photocatalysts for solar photovoltaic applications [35,36]. In addition to this, atomic layer deposition (ALD) is also a promising approach to forming inorganic matrices or coatings to encapsulate QDs. Studies have demonstrated that ALD growth can protect QDs against environmental degradation, oxidation, ripening, and sintering [37,38]. Moreover, ALD growth can enhance the performance of QDs and QD layers, which will result in increased charge carrier mobility and charge carrier multiplication [1,5,39]. As a result, the ALD process has enormous potential as a method for post-functionalizing QDs in addition to encapsulating and protecting them from oxidation. In this work, we developed a novel approach to the Al_2_O_3_ capping of PQDs by using ALD. The experimental results show that Al_2_O_3_ capping can indeed improve reliability while maintaining efficiency. 

## 2. Materials and Methods

In this study, a PQD film with high reliability was developed. First, see Chen et al.’s synthesis of FAPbBr_3_ QDs [40]. FAPbBr_3_ QDs were synthesized using the ligand-assisted re-precipitation (LARP) method at room temperature [40,41]. First of all, 500 μL of oleic acid (OA), 0.16 mmol formamidinium bromide (FABr), 0.2 mmol lead (Ⅱ) iodide (PbBr_2_), and 20 μL of octylamine were mixed. After thoroughly mixing, 10 mL of toluene was gently added and stirred for 5 min, and then 5 mL of acetonitrile was added, followed by mixing uniformly. High-speed centrifugation was used to separate the mixture in the solution for 25 min at 6000 rpm, after which the supernatant was removed, and the pellet was then dissolved in 10 mL of toluene. After uniform mixing, 30 μL of an excess-lead-ion solution formed by mixing 1.512 mmol PbBr_2_, propionic acid, butylamine, and n-hexane was added. Then, 500 μL of OA and 10 mL of methyl acetate were added to the solution, allowed to react for 5 min, and then centrifuged for 25 min at 8000 rpm. After removing the supernatant, the pellet was once again dissolved in 5 mL of toluene and centrifuged at 8000 rpm for a further 15 min. Finally, a clear green liquid, i.e., the FAPbBr_3_ PQD solution, was collected, which was ready for post-synthesis surface optimization [42,43]. Next, the Atomila GT atomic layer powder spraying equipment developed by SkyTech Institute (Hsinchu, Taiwan) was used to coat the surfaces of PQDs with Al_2_O_3_ to realize the passivation protection of PQDs. A protective layer of Al_2_O_3_ was deposited on PQDs using trimethylaluminum (Al(CH_3_)_3_, TMA) and ozone (O_3_) as precursors and water as a co-reactant at 150 °C for 200 cycles (deposition rate: 2.5 Å/cycle), and the resulting sample is named PeQD. The ALD equipment developed by SkyTech Institute has a special powder “dust flow field” design with a cavity that can rotate 360 degrees to generate a uniform powder flow field. It is different from the previous plating on the surface of the QD film with a protective layer [39,44,45,46,47,48]. The method proposed in this study can improve the application degree of QDs. Finally, we mixed PeQDs, KSF red phosphor, nanoscale TiO_2_ scattering particles, dispersant, and UV glue uniformly for PeQD thin-film preparation. The mixture was poured into a model, and air bubbles were removed from it using a vacuum pumping system. Finally, UV light was irradiated to cure it, and then the PeQD thin film was formed.

## 3. Results and Discussion

### 3.1. Surface Topography Analysis

**Figure 1:**
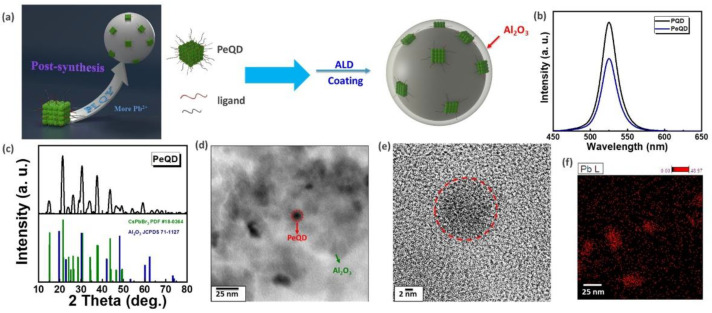
Surface topography analysis of PeQDs. (**a**) Schematic diagram of surface modification. (**b**) Spectra of PQDs and PeQDs. (**c**) XRD image. (**d**) TEM image. (**e**) High-magnification TEM image. (**f**) Mapping analysis image.

The instability of PQDs’ surface bonding is well understood. When QDs are subjected to external stimuli, such as light, hot water, oxygen, etc., the surface ligands are easily peeled off, resulting in the phenomenon of fluorescence quenching and causing a significant drop in the photoluminescence quantum yield (PLQY) [49,50]. This study referred to Chen et al.’s ligand-assisted re-precipitation (LARP) method for synthesizing QDs [40]. The aggregation phenomena of QDs and surface defects of QDs induced by the dissolution crystallization method were monitored using post-synthesis optimization processing technology. In this method, excess Br^-^ is added to the purified FAPbBr_3_ QD solution to make up for the vacancy of halogen anions generated during the purification process and to increase the bonding strength between the ligand and the QD surface [42]. Then, the ALD passivation protection technology is used to coat the surfaces of the PQDs with oxides, which are named PeQDs. This passivation layer not only protects the QDs from high temperatures and any changes in material properties but also protects the QDs from moisture and oxidation. This is an effective passivation method at present, and a schematic diagram is shown in Figure 1a. Figure 1b shows a comparison of the optical properties of post-synthesis optimization processing technology and ALD passivation protection technology. PeQDs and PQDs have wavelengths, full width at half maximum (FWHM) values, and PLQYs of 525, 25 nm, and 99% and 525, 25 nm, and 78%, respectively. After applying ALD passivation protection technology, there is a slight decrease in the PLQY part. This is attributed to the addition of water and high temperature during the coating process. Figure 1c shows the XRD analysis. It can be observed in the figure that the PeQDs do not produce any phase changes, indicating that the ALD passivation protection technology does not affect the QD structure. Figure 1d,e present the TEM images of Al_2_O_3_ coated on the surfaces of PeQDs. The gray portion in the figure is the Al_2_O_3_ matrix, and the darker spots correspond to PeQDs, which is confirmed by the mapping analysis shown in Figure 1f. The ALD passivation technology developed in this study was used to coat the surfaces of PeQDs with Al_2_O_3_, which can effectively isolate the influence of water vapor and oxygen, thereby improving reliability.

### 3.2. Optical Characterization Analysis

**Figure 2:**
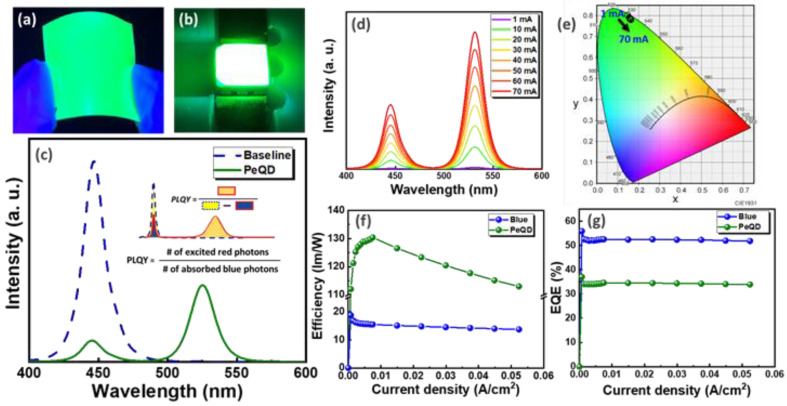
Optical properties of green PeQD film. (**a**) UV lamp, (**b**) light color image of blue LED excitation. (**c**) Emission spectrum (20 mA). Current variation test (1–20 mA) (**d**) emission spectrum image, (**e**) CIE color coordinates, (**f**) luminous efficiency, and (**g**) EQE.

Green PeQDs treated with ALD passivation were uniformly mixed with UV glue to make composite films, and their optical characteristics were evaluated. Figure 2a,b show the optical illumination from the PeQD composite film under UV light and blue LED exposure, respectively, showing a bright green-colored light. The PeQD film under 446 nm blue LED exposure exhibits an emission wavelength of 525 nm, an FWHM of 25 nm, and a PLQY of 45%. As shown in Figure 2c. PLQY is defined as the ratio of the emitted photons to the absorbed photons as follows [51]:

(1) $$\mathrm{PLQY}=\frac{Emission}{Absorbance}=\frac{\#\;of\;excited\;greem\;photons}{\#\;of\;absorbed\;blue\;photons}$$

The driving current for the blue LED was changed from 1 to 70 mA in order to test the reliability of the PeQD film. Figure 2d,e illustrate spectrograms and the Commission Internationale de l’Eclairage (CIE) color coordinates of the PeQD film, respectively, with a varying driving current. It can be seen that the Al_2_O_3_ layer can indeed effectively protect PeQDs from the high radiation of the blue LED illumination. With the increasing current, the CIE color coordinates are slightly shifted from (0.147, 0.803) to (0.163, 0.781), with no emission wavelength shift. The PeQD film exhibits a maximum luminous efficiency of 130.4 lm/W at a current density of 7.48 × 10^−3^ A/cm^2^ (10 mA) and a minimum luminous efficiency of 112 lm/W at a current density of 7.48 × 10^−4^ A/cm^2^ (1 mA), as shown in Figure 2f. EQE is defined as the ratio of the number of photons emitted into free space per second to the number of electrons injected into the LED per second, where P_0_ is the radiant power (coupled to free space), hν is the photon energy, I is the injection current, and e is the fundamental charge, and is calculated as follows [52]:

(2) $$\mathrm{EQE}=\frac{Emitted\;photons\;outside\;LED/s}{Input\;electrons/s}=\frac{P_{0}\;/\;h\nu }{I/\;e}$$

The external quantum efficiency (EQE) of the PeQD film is 37.0%, as shown in Figure 2g, showing an EQE stability as a function of current density. The experimental results confirmed that the green PeQD film treated with ALD passivation protection can effectively improve reliability. In addition, the PeQD film displayed no emission wavelength shifts with the changing current and excellent light-emitting characteristics, which is promising for future optical applications.

**Figure 3:**
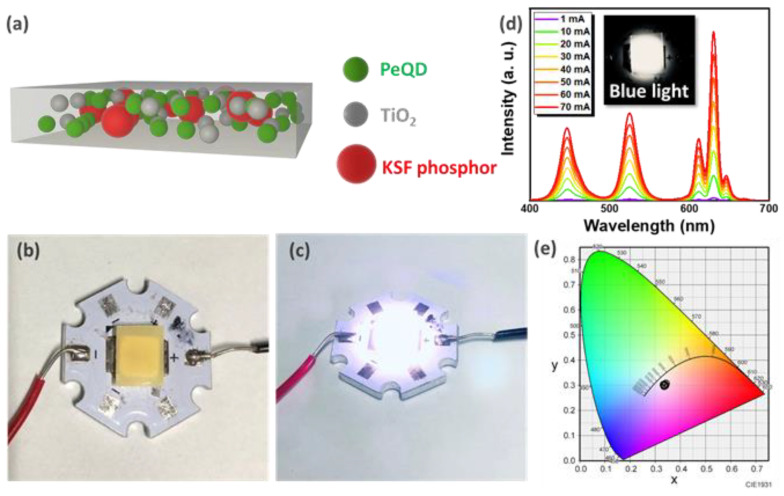
Optical properties of white PeQD composite film. (**a**) Schematic image of film formation. (**b**,**c**) Optical image of white-light LED (20 mA) with and without illumination. Current variation test (**d**) emission spectrogram and (**e**) CIE color coordinates.

With the research findings in the above section, it is essential to develop white-light components to increase the potential application of green-light PeQD powders. The PeQDs were uniformly mixed with KSF red phosphors having an emission wavelength of 630 nm and UV glue and then excited by blue LEDs (446 nm) to make white LEDs. This strategy, however, is ineffective since the reabsorption effect is caused by the self-aggregation of the nanoscale PeQD powder [5,24,29,53]. The light scattering is enhanced by adding a light scatterer, i.e., TiO_2_ nanoparticles, to the mixture. By mixing TiO_2_ nanoparticles in PeQDs, KSF red phosphor, and UV glue, the optical path direction of blue light can be increased and can become randomized, thereby increasing the absorption of blue light by PeQDs [23,24,54] and improving the light conversion efficiency (LCE) of PeQDs. A schematic diagram for the composite film consisting of PeQDs, TiO_2_, and red phosphor is shown in Figure 3a. Figure 3b,c show the optical images of the white-light devices with and without illumination. Figure 3d,e show the emission spectrum and CIE color coordinates of the white-light device with the increasing current. The emission spectra of the white-light device, including the three primary colors, R, G, and B, exhibit no wavelength shifts with an increase in current. The chromaticity coordinates of the device are only slightly shifted from (0.34, 0.31) to (0.34, 0.30). This is because the surfaces of PeQDs are fully passivated with ALD technology, which can effectively mitigate the impact of light radiation and significantly increase reliability, which is highly beneficial for market demands. 

### 3.3. Reliability

**Figure 4:**
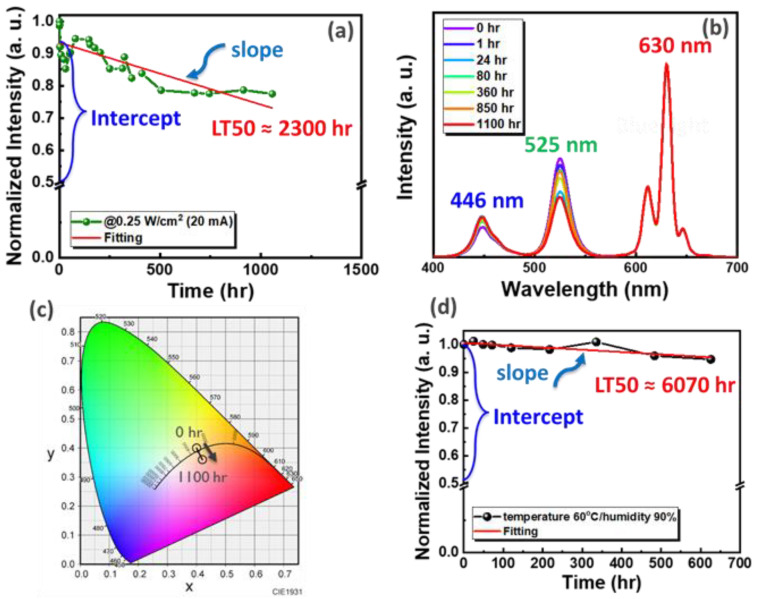
Reliability test of white PeQD composite film. White PeQD composite film (20 mA) excited with blue light: (**a**) decay trend image, (**b**) emission spectrum, and (**c**) CIE color coordinates. (**d**) Aging test at 60°/90%.

Although PQDs have excellent optical properties, the reliability issue is the key to their application. To significantly enhance their PLQY, PeQDs first undergo post-synthesis optimization processing technologies to passivate PeQD surface defects. Then, ALD passivation technology is introduced to protect the QDs from oxidation, moisture, high temperatures, and any changes in the material characteristics. Figure 4a shows the reliability test of the white PeQD composite film, where the long-term lighting test was carried out using a blue LED, with the current being fixed at 20 mA. It was found that after long-term lighting for 1100 h, the PLQY only dropped to the original 0.225 compared with PQD films without ALD passivation protection treatment. Due to the lack of Al_2_O_3_ protection, the original PLQY of the PQD film was reduced to 13%. After the long-term aging test, the PLQY of PQDs was lower than the original 50% within 12 h. This shows that ALD passivation protection technology can effectively improve the reliability of QD films. In order to properly assess the long-term lighting lifetime of a component, a criterion called LT50 can be linearly extrapolated from the last 1100 h of data, defined as the lifetime to 50% of its initial value, and 50% of the initial peak intensity is calculated as follows [29]:

(3) $$\mathrm{LT}50=\frac{\left(50\;-\;intercept\right)}{slope}$$

where the slope and intercept are linear fit parameters to the data we obtained during the successive aging of each device.

The long-term lighting reliability spectrum is shown in Figure 4b. Its CIE color coordinates are shifted from (0.40, 0.40) to (0.42, 0.36), as shown in Figure 4c, showing excellent reliability. In order to explore the influence of temperature and humidity on PeQDs, a long-term test with temperature and humidity of 60°/90% was carried out, and after 624 h, the attenuation was only 0.053, as shown in Figure 4d. These results indicate that ALD passivation technology can effectively protect QDs from high temperatures and any changes in material properties and protect QDs from moisture and oxidation.

### 3.4. Visible-Light Communication (VLC)

**Figure 5:**
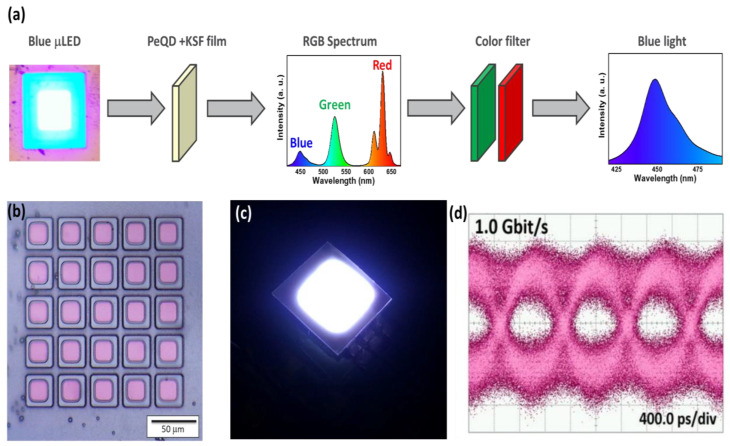
White PeQD composite film VLC application. (**a**) Architecture image. (**b**) Optical microscope (OM) image of 50 μm micro-LED. (**c**) Light color diagram of blue micro-LED combined with white PeQD composite film. (**d**) Detected NRZ-OOK eye diagram at 1 Gbit/s.

In order to create efficient full-color displays and white LEDs for high-speed visible-light communication, it has become increasingly common in recent years to integrate micro-LEDs and colloidal QDs as color conversion layers. The VLC application architecture image is shown in Figure 5a [55]. We used a 30 μm sized micro-LED array as the light source in the data transmission experiment, as shown in Figure 5b. Figure 5c shows the data transmission characteristics of a blue micro-LED combined with the white PeQD composite film analyzed by an on–off keying (OOK) system. The test bit sequence is a non-return-to-zero (NRZ) 2^7^-1 pseudo-random binary sequence (PRBS7) generated by a bit pattern generator (Anritsu MP1800A). The frequency response of the white PeQD composite film and μLED was measured after being passed through an optical filter, and the −3 dB bandwidth was 500 MHz at an injection current of 10 mA (current density of 35 A/cm^2^). Figure 5d shows the detected NRZ-OOK eye diagram showing a clear opening of the eye diagram at 1 Gbit/s, indicating the potential application of the micro-LED combined with the white PeQD composite film for high-speed VLC applications.

## 4. Conclusions

In summary, we demonstrate a technique for coating oxides on the surfaces of QDs using ALD passivation protection technology. With this method, QDs can be effectively protected from high temperatures and any changes in material properties and can also be protected from moisture and oxidation. The PeQDs exhibit significant wavelength stability with a change in current. The green PeQD film treated with ALD passivation has a maximum luminous efficiency of 130.4 lm/W. Green PeQDs, KSF red phosphors, and dispersive TiO_2_ particles were mixed into the process film, and the blue LED was used to excite the composite film to make a white LED. After 1100 h of the long-term aging test, the efficiency only dropped to the original 0.225 with an LT50 of about 2300 h, and for the 60°/90% temperature/humidity test, LT50 was about 6070 h. The white-light device made by using the PeQD composite film exhibited a high data transmission rate of 1 Gbit/s. These results suggest that PeQDs protected by ALD passivation are very promising for full-color display and VLC applications.
